# Dogs on Film: Status, Representation, and the Canine Characters Test

**DOI:** 10.3390/ani14223244

**Published:** 2024-11-12

**Authors:** Nicole R. Pallotta

**Affiliations:** Animal Legal Defense Fund, Portland, OR 97214, USA; npallotta@aldf.org

**Keywords:** animal status, film studies, representation, law and culture, multispecies family, animals in film, canine characters, human–dog relationships

## Abstract

The representation of animals in cinema and television reflects and reinforces dominant ideologies and traditional stereotypes. While popular culture often legitimizes prevailing social norms and existing power relations, it can also reflect shifting cultural attitudes about traditional axes of inequality such as race, gender, class, sexual orientation, disability, and species. Representations of canine characters incidentally embedded in family life provide a unique lens through which to consider the evolving cultural and legal status of dogs and their place in multispecies families. This article introduces a Canine Characters Test, similar to the Bechdel Test used to measure the representation of women in movies, to critically evaluate the representation of dogs in film and television. Applying the test and its four criteria—Role in Narrative, Agency, Language, and Animality—to two examples, this article argues that portrayals that pass the test support a positive shift in social norms regarding dog–human relationships, which in turn bolsters efforts to elevate dogs’ status under the law. Benevolent speciesism, authenticity, and problematic tropes such as vanishing, ornamental, and miraculous canines are also discussed.

## Simple Summary

This article introduces a Canine Characters Test, similar to the Bechdel Test, to critically evaluate the representation of dogs in film and television. Drawing on concepts of benevolent speciesism and authenticity, this article argues that portrayals that pass the test support a positive shift in social norms regarding dog–human relationships, which in turn can improve dogs’ status under the law.

## 1. Introduction

Works of popular culture, including movies and television, both reflect and construct idealized social relations and norms, and can reinforce stereotypes pertaining to gender, race, class, sexual orientation, disability, and animality. I use the term animality here to refer to nonhuman animals “as they are”/in themselves—as opposed to how humans (often inaccurately) perceive them to be based on stereotypes, speciesism, and lack of information [1,2]. 

In their core treatise on critical theory, Horkheimer and Adorno [3] argued that what they called the “culture industry” (including films) reflects social reality as it is, lulling viewers into passivity and acceptance of dominant ideologies. Later cultural theorists would emphasize the dynamic interplay between viewer and text, recasting consumers of film and other mass media as active participants in meaning-making, and pop culture as an arena of negotiation and resistance [4].

While pop culture products are often regressive and telegraph messages that reinforce the status quo, they also grapple with, and can be harbingers of, progressive social change, portraying possibilities for new social relations—sometimes overtly and sometimes obliquely. For example, trends in romance films during the twentieth century reflected changing attitudes about race, ethnicity, and social class. The erosion of status conflicts that were once considered insurmountable barriers created a loss of dramatic tension, which resulted in a shift from the predominance of the romantic drama to romantic comedy [5]. Yet in this genre, even as status conflicts dwindled, the representation of gender has remained problematic [6,7,8,9].

Feminist theorists, particularly those identifying as eco-feminists, have long drawn connections between the oppression of women and the exploitation of animals [10,11,12,13]. Parallels can be found in cinematic criticism as well. Like the “male gaze”, which is rooted in patriarchal society and depicts women from a masculine, heterosexual perspective as sexual objects for the male viewer’s pleasure [14], animals in film are subject to the human gaze [15], which is grounded in anthropocentric society.

Popularized in the 2000s, the Bechdel Test has entered the popular lexicon as a rough measure to evaluate the inclusion and representation of women in film [16]. Its modest threefold criteria are that a movie has (1) at least two women in it (2) who talk to each other (3) about something besides a man. The fact that so few movies could pass this minimal test illuminated the extent of gender disparity in Hollywood cinema. Critics have extended the Bechdel Test to television, books, and other media, and similar tests have been developed to measure representation of other marginalized groups [17,18,19,20].

In this article, I introduce a Canine Characters Test to evaluate the representation of dogs in film and television. This discussion is framed by the ambivalence between dogs’ legal status as property and an emerging cultural understanding of them as family members.

In linking popular culture, like film and television, to issues of law and policy, I situate my analysis within the critical theory tradition of taking mass culture seriously as a medium that both mirrors and constructs idealized social relations and norms. While popular culture often legitimizes prevailing social norms and existing power structures, it can also reflect and support shifting cultural attitudes about traditional axes of inequality—including species—and thus has the ability to challenge dominant ideologies about human–animal relations.

## 2. Dogs on Film

Numerous theoretical questions arise when we consider the representation of animals across a wide variety of film and television genres—from animated cartoons to realistic documentaries [15,21,22,23,24,25,26,27,28]. However, such a broad-ranging discussion is beyond the scope of this article. Here, I narrow the focus to animals culturally classified as companions, and more specifically to dogs due to their integration into our homes and lives as quasi-family members.

I use the word “quasi” here because while many individuals consider their dogs to be family members and treat them as such, nonhumans are not typically recognized within legal definitions of “family” in the U.S. For example, “familial status” under the federal Fair Housing Act includes children but not companion animals. Throughout the rest of the article, I will refer to dogs as family in the colloquial, nonlegal, use of the term.

I am also primarily interested in works that depict dogs as secondary or tertiary characters woven into the life of the family. While the Canine Characters Test can be applied to any type of portrayal, productions in which a dog is the main protagonist present qualitatively distinct questions.

Thus, the examples discussed here, like countless other films and TV shows, do not revolve around the dogs. Yet creators made the interesting choice to include them in the narrative as occasional characters—more than extras but less than main protagonists. Representations of canine characters incidentally embedded in family life provide a unique lens through which to consider the evolving cultural and legal status of dogs and their place in multispecies families.

### 2.1. The Liminal Status of Companion Animals in Society and Law

Companion animals, including dogs, currently occupy a liminal place in society and law, due to the tension between a growing private understanding of them as family and their classification as property in the public realm of the legal system. While property status itself does not preclude recognition of expanded legal rights, even within the private sphere of the home there is vast inconsistency, with some people treating their companion animals as cherished family members and others treating them like expendable objects—which is more in line with the property classification. Although a family member understanding is gaining ground, social forces, including the law, are in conflict, with some bolstering an emergent respect for companion animals as individuals with inherent value, and others reinforcing an antiquated notion that they are disposable commodities with minimal intrinsic worth [29].

In order to resolve this ambivalence, social norms must change. However, changing the law can in turn help shift social norms, amplifying some norms over others when a discrepancy exists. Though law does not determine culture, when competing social norms are in conflict, the law can help resolve tensions [30,31]. This expressive or symbolic function of law—in contrast to its substantive function of providing rights, duties, and sanctions—serves to shape group values and norms, which in turn influence individual attitudes [32]. Some research has shown that even laws without sanctions, or which are unenforced, can influence behavior [33,34]. Popular culture serves a similar function during periods of social change—helping to crowd out some norms and amplifying others.

### 2.2. Dogs as Family?

Many depictions of dogs in film, and other media like advertisements, present an idealized version of the family member narrative. Such portrayals stand in stark contrast to the reality for many dogs who endure lives of quiet neglect, whether confined alone in a house all day or forced to live on a chain in the backyard. Much of this neglect is culturally invisible and legal, as animal cruelty laws in the U.S. generally address only minimum physical survival needs (such as food, water, shelter, and sometimes basic veterinary care), but not higher-order behavioral, social, and psychological needs like exercise, companionship, and mental stimulation.

In the context of routine mistreatment of companion animals—whether through maliciousness, carelessness, or ignorance of their needs—such sanitized portrayals serve a hegemonic function, supporting a capitalist economic system and sustaining the multibillion-dollar-per-year pet industry. As Pierce notes:

The pet industry preys on our love for animals and exploits it…. through cultivating a cultural narrative in which pet keeping is part of a normal and happy life and in which a complete family includes at least one nonhuman member. Within this narrative, pets are loved and cherished and treated with tenderness, just like children. Thus we are told, over and over: nine out of ten pet owners consider their pet a part of the family. Why does this statistic get repeated so often? It is almost like an advertisement for pet keeping. Oh, wait. It *is* an advertisement.

[35] (p. 179)

This oft-heard statistic can be traced back to pet industry trade groups [35], and despite frequent repetition in the media, survey data regarding companion animals as family are flawed for several reasons [29]. Cinematic narratives that uncritically present dogs as respected family members support a cultural myth that fuels an entire industry, but does not serve dogs themselves or advance their place in society.

Rather, this myth is an instrument of social reproduction in that it distracts from real material conditions, in which dogs as a class—no matter how well an individual may be treated—are vulnerable to mistreatment and abandonment, in part due to their status as personal property. Structural reasons for this vulnerability include a lack of meaningful representation in the legal system coupled with, and as a result of, the dominant ideology of speciesism, which justifies their treatment as legal objects versus subjects.

This lack of meaningful representation stems from the substantive shortcomings of animal protection laws, underenforcement of laws that do exist, and perhaps the biggest obstacle of all: antiquated and anthropocentric standing requirements that routinely exclude not only animals but also their human advocates from the courtroom and hence access to justice. Property status, without attendant robust species-specific rights, will always trump benevolent notions of family, which are dependent on the kindness of individuals rather than systemic justice, which would clearly codify this status in law and public policy.

Alternatively, one might argue that narratives glorifying the virtues of dogs and their role in the emotional sphere of the family serve a positive function. Though this may not be the reality for a majority of dogs in the U.S., it communicates a utopian vision of an alternate social reality that may be on the horizon. Rather than serving a hegemonic function, depicting idealized relations may help usher in new and better social conditions by providing alternative narratives that can help to change collective consciousness. Yet, even here, it is important that dogs are presented as the animals they are, with species-typical behavior and needs.

### 2.3. Characters Versus Props

A related question arises when we consider the representation of dogs on film: are they better understood as characters or props? Hanks argues that since the end of the silent film era, dogs in film have functioned as props, “largely confined to a series of symbolic functions … they are reduced to foils for human stars” [36] (n.p.). Horowitz likewise writes of this tendency: “Dogs are used for their generic roles—as props, as part of the scenery, as part of the family—but are not considered as dogs, as individual animals” [37] (p. 231).

Hanks [36] notes that in recent years, two notable trends have emerged: the “tiny yappy dog as comic prop” and the dog as victim, where a dog is killed on-screen for shock effect. Websites like www.moviepaws.com and www.doesthedogdie.com track the deaths of canine characters in film. Film critic Joe Queenan observed that animals are “the final frontier of convenient victims, especially since they don’t have voices to protest such depictions” [38] (n.p.). He posits that even if animals are not harmed in making these movies or shows, “the violence on screen can affect how the public views animals. As it is, many people still view animals as disposable property, and often abuse, abandon, or dump pets at the shelter daily” [38] (n.p.). Neither of these tropes grants dogs sufficient agency, rooted as they are in metaphor—with dogs serving as symbols rather than subjects—and tied to the human gaze.

It is important to note that the human gaze refers not to the fact that humans are literally the ones behind the camera telling the story, but to the way in which animals are portrayed—as subjects with agency or as anthropomorphized or objectified props. Similar to how the female gaze inverts patriarchal depictions of women, an animal-centric gaze portrays animals as characters and subjects in their own right rather than props, ornaments, or metaphors for human struggles or societal issues. Hanks argues the following:

The only director who seems prepared to grant a dog real autonomy and complexity is Jean-Luc Godard: in *Goodbye to Language* (2014), Roxy, Godard’s own dog, isn’t entirely freed from a traditional symbolic role…. But as Roxy snuffles about in woods and a river—the instinctual animal—he also seems to have a whole life…. For once, it feels as though we’re seeing a real, self-sufficient dog.

[36] (n.p.)

The word “prop”—referring to objects used on-screen or stage by actors during a performance—is an abbreviation of “property”. An interesting parallel can be drawn between animals’ legal status and their status in film. Though debates about animals’ legal status are often framed as property *or* legal personhood, these categories are not mutually exclusive: an entity can be both. For example, corporations and ships can be both property and persons for legal purposes, which highlights the fact that these categories are not binary but can overlap. Children, to whom dogs are sometimes compared, were themselves considered property of their fathers throughout much of history [39].

Just as an animal can be both property and a legal person, so the categories of prop and character are not mutually exclusive. These categories are situated on a continuum, and where an animal falls on it depends on their role in the story being told and how they are depicted. Determining whether dogs on film are more accurately described as props or characters depends on the production in which they appear and whether they pass the Canine Characters Test.

## 3. Benevolent Speciesism and Authenticity

The Bechdel Test illuminated the frequency with which mainstream movies represent women primarily in relation to men. These one-dimensional portrayals reflect patriarchal ideology and sexist stereotypes that minimize female characters’ subjectivity and complexity. Likewise, dogs have their own subjective experiences that are flattened through portrayals that reinforce speciesist stereotypes. Similar to what has been called “benevolent sexism” [40,41], these stereotypes at first glance may not appear harmful to dogs because they are positive.

Hostile speciesism may include beliefs that all dogs (or all individual dogs of a certain breed) are aggressive, stupid, dirty, or destructive. These negative stereotypes can give rise to policies that are harmful to dogs and people alike, such as breed-discriminatory legislation and blanket “no pets” policies in rental housing.

In contrast, benevolent speciesism may include ideas about how “wonderful” dogs are—similar to the “women are wonderful” effect in which women are regarded positively but paternalistically, and receive protection and affection for conforming to traditional gender roles. Individual dogs may be wonderful but, as noted by Glick and Fiske, “subjectively positive stereotypes are not necessarily benign” [42] (p. 109). For example, all Asian-Americans are intelligent, hardworking, and good at math is a positive, but racist, stereotype that contributes to the harmful “model minority” myth that flattens individual differences as well as diversity of experience across ethnic groups [43].

Together, benevolent and hostile sexism comprise “ambivalent sexism” [42]. This conceptual framework has been applied to classism [44], ageism [45], and ableism [46], and can be applied to speciesism as well.

### 3.1. Unconditional Love?

Positive stereotypes applied to dogs often highlight the esteem in which dogs supposedly hold humans (the aspirational quote “be the person your dog thinks you are” being but one example). This stereotype objectifies dogs by treating them as if they were unconditional love dispensers. Suggestive of an anthropocentric narcissism, the wonderful qualities that dogs are thought to possess often reflect what dogs do for us and how they make us feel—not who they are. Dogs are often exalted because of how well they seem to exalt humans. Yet dogs have their own experiential worlds and subjective experiences apart from their role as mascots in human lives.

(Interestingly, the Spanish word *mascota* translates to both “pet” and “mascot”. Both definitions are derived from the French *mascotte*, referring to a lucky charm or talisman.)

Benevolent speciesism also highlights qualities that purportedly make dogs “better” than people, especially, as noted above, their steadfast loyalty and capacity for unconditional love. While it may sound like a positive attribute—and is intended as such by those who bestow it—the concept of unconditional love as it is applied to dogs is problematic [47]. It ignores power dynamics and the fact that dogs in the U.S. are dependent upon their legal owners for everything, from basic necessities like food, water, and shelter, to higher-order social, behavioral, and psychological needs like companionship, exercise, and mental stimulation. And there is tremendous disparity in how dogs are (legally) treated in the U.S. As Irvine notes: “Within our household, the animals are not considered property. However, outside of the household, that is exactly their status. I am free to pamper them or ignore them, as long as I am not caught inflicting intentional cruelty” [48] (p. 14).

In this context—a relationship where one member is the property of the other and dependent upon them to meet all of their needs—unconditional love must be viewed critically, if not cynically. Shifting the focus from unconditional love as something dogs are expected to provide their owners and placing this expectation instead on human caregivers puts the relationship on more equitable footing. For people to love their dogs unconditionally—meaning being committed to their lifetime care and attentive to their species-typical needs—mitigates the power imbalance inherent in dog–human relationships. This power differential is reflected in the fact that, love notwithstanding, dogs are the legal property of their owners.

### 3.2. Objectification and Anthropomorphic Infantilization

Other positive stereotypes are rooted in anthropomorphism. While anthropomorphism may seem harmless at best and annoying at worst when applied to dogs, in fact it can be just as harmful as its counterpart: objectification. Objectifying dogs (and other animals) means treating them more like inanimate things than complex, sentient beings. Related is anthropodenial, or “the a priori rejection of shared characteristics between humans and animals when in fact they may exist” [49] (p. 258). While de Waal cautions “we must be very careful not to exaggerate the uniqueness of our species” [50] (p. 51), anthropomorphism errs at the other extreme—treating dogs as if they were human.

The charge of anthropomorphism has historically been a moving target, as many traits and abilities once believed to be the sole domain of humans are now known to exist in other species, with new understandings of animal behavior and cognition aided by technological advances and a shift in perspective acknowledging animal agency [51]. Without denying that many nonhuman animals share some traits and capacities with humans, they also have their own species-typical needs and capabilities that can be obscured through anthropomorphism.

De Waal differentiated between “animalcentric anthropomorphism”, which can be useful as a heuristic tool and to develop testable hypotheses, and “anthropocentric anthropomorphism”, which presents talking animals and other cartoonish depictions of animals that have little or nothing to do with their actual attributes but rather “serves human social purposes: to mock, educate, moralize, and recreate” [49] (p. 261).

Animalcentric anthropomorphism is “…a more mature anthropomorphism, in which the human perspective is replaced, however imperfectly, by the animal’s” [49] (p. 262). Anthropomorphism “comes in many shapes and forms” and is not always negative, ranging “from the naive projection of human experience onto other species to serious attempts to understand animals on their own terms through intimate familiarity with their behavior and Umwelt” [49] (p. 273).

What I am calling “anthropomorphic infantilization,” or treating dogs as if they were human children, denies their animality and umwelt, or subjective sensory experience of their world [52]. This is where the harm lies in treating dogs as if they were “children in fur coats” or “fur babies”. Those who call their dogs “fur babies” likely mean it as a figurative, not literal, term of endearment. However, it points to a wider trend that is real [53,54,55].

People who anthropomorphically infantilize their dogs may love them but lack awareness about dog behavior and needs, thus overlooking what they need to flourish rather than just exist. As dog behaviorist and trainer Ross McCarthy notes:

We cause problems because it is all about us—not all about them. They have to fit into our lives, without us making any effort to understand theirs…. Dogs have survived for centuries just fine without wearing clothes, being carted around in some kind of case, being imposed on by people who believe that they should always behave like fluffy little people in polite society.

[55] (n.p.)

In addition to denying dogs’ umwelt, treating them like “fluffy little people” can lead to unrealistic expectations that cause negative outcomes, including aggression when dogs are forced into situations in which their distress or fear goes unrecognized by their caregiver due to lack of awareness about canine communication. When dogs fail to act like the children they are expected to be, without species-appropriate training and guidance, they are at risk for ejection from the family unit. When people relinquish their dogs to shelter facilities, behavioral issues are consistently among the top reasons given during intake [56].

### 3.3. Being a Dog

The Bechdel Test underscored the fact that portrayals of women in mainstream movies often reduce them to support for male characters and, in contrast, promoted the idea that “the women on TV and in movies ought to be characters, not cliches” [57] (n.p.). Positive stereotypes likewise confine dogs to roles supportive of humans. Like the male gaze centers men as subjects and objectifies women, so does the human gaze objectify animals by portraying them as props, symbols, or caricatures. Freeman and Merskin note that animals’ real lives become invisible when they are rendered symbolically:

As a result, they are even more vulnerable, particularly when they are presented as comic fodder or used as symbolic stand-ins for human emotions in greeting cards, comic strips, commercials, and multi-media content. Rather than bringing us closer to understanding, which is what healthy levels of anthropomorphism can do, these representations further distance them from us.

[58] (p. 209)

In contrast, an animal-centric gaze depicts caninity similar to the way the female gaze presents women as multidimensional and complex human beings.

The most important right for any animal in human custody is arguably the right to be the animal they are, i.e., to express natural behaviors and receive species-appropriate care. The Animal Legal Defense Fund encapsulates this sentiment in the fifth tenet of its aspirational list of six essential rights for animals: “The right of animals under human care to have their species-typical and individual needs fulfilled to maximize their physical, emotional, and mental well-being” [59] (n.p.). These criteria are also reflected in the Five Domains Model for assessing animal welfare [60,61].

When considering what animals need to thrive, it is important to adopt their perspective. Regarding the importance of treating dogs like dogs, Houston summarizes Paul McGreevey and Alexandra Horowitz, experts in dog cognition and behavior, thusly: “What [they] are both saying, in essence, is instead of imagining that your dog is human, imagine what it might be like if you were the dog” [54] (n.p.).

## 4. The Canine Characters Test

Inspired by the Bechdel Test, the Canine Characters Test evaluates the representation of dogs in film based on four criteria that weave together the above threads—benevolent speciesism, objectification, anthropomorphism, and the importance of being a dog.

Like the Bechdel Test, a primary theme of the Canine Characters Test is authenticity, because representation matters and affects cultural opinions, attitudes, and beliefs [22,23,24,62,63,64,65]. Representation matters outside of film as well. Literature, advertisements, and children’s books and toys are just a few of the other important contexts in which dogs are represented that influence culture and ideology. While this article and the Canine Characters Test are limited to representations in movies and television, the test could potentially be adapted to other media and other genres within film besides those featuring dogs as family members and secondary characters. Preliminary questions to be included in the test are presented below:

1. **Role in Narrative:** Does the dog figure prominently in the main story or subplots? Do they direct the action in a meaningful way? Is the dog integral to the story and the family, or do they serve a more ornamental function?
2. **Agency:** Does the canine character have an opportunity to display agency? Is the relationship between the dog(s) and human(s) characterized by mutual respect and cooperation, laissez-faire noninterference, or domination and control?
3. **Language:** Does the dog have a name? Are they referred to as “who,” “s/he,” or “they” or with pronouns reserved for inanimate objects, such as “it” or “that?”
4. **Animality:** Does the canine character act like a dog? Are they portrayed naturalistically in a way that reflects their species-typical nature, or are they anthropomorphized or objectified?

The Bechdel Test’s simple criteria highlighted that women’s role in the narrative is often ornamental, serving or supporting male characters and providing visual fodder for an objectifying male gaze. Dogs may also be depicted in ways that objectify them, as props, ornaments, or symbols rather than characters in their own right.

These criteria, like those provided by the Bechdel Test, are intended to provide a minimum baseline for authentic/animal-centric representation and not be the endpoint for analyses of the portrayal of family dogs in film and television. The fact that a film passes the Bechdel Test does not necessarily mean it has a feminist message, and vice versa. Similarly, the Canine Characters Test is intended to provide a floor, not a ceiling, for authentic/non-speciesist representation. These criteria can also be expanded and revised to apply to other types of animals; for example, to evaluate representations of farmed animals or wild animals in film and TV. In addition, these criteria can be used to inform debates about the legal and cultural status of animals as reflected in, and constructed by, works of popular culture.

These criteria reflect factors that are important to the well-being of dogs: the opportunity to exercise agency and choice (Agency) and to be a dog (Animality). The ability to exercise agency is critically important for animal well-being [60,66,67,68]. Yet, the lives of family dogs (as opposed to free-ranging or community dogs) are heavily circumscribed. The opportunity to exercise choice and control, and to signal consent (e.g., to be petted), is crucial and often unrecognized or ignored (this is easily observable by anyone with a modicum of knowledge about dog behavior in common human–dog interactions on the street, at the dog park, or in other public settings).

As discussed above, a harmful stereotype is that “good” dogs know how to behave according to social norms and human expectations in social settings and at home. Particularly without positive training, guidance, and socialization, it is challenging for dogs to know what is expected of them in a human world. This lack of cross-species understanding can lead to dogs being rigidly controlled, left out of activities because they are “poorly behaved”, or abandoned or rehomed. Respect for domestic dogs’ animality, including providing ways to exercise agency, is a cornerstone of integrating them into multi-species families and human society in ways that benefit everyone in the situation.

Being a dog means neither being anthropocentrically anthropomorphized [49] nor, on the other hand, being objectified or referred to using words reserved for inanimate objects (Language). The changing role of animals in society and their increasing importance in the family are reflected on-screen through their representation as characters versus props (Role in Narrative).

These criteria also reflect areas in society and law where speciesist stereotypes harm dogs. Objectification in cinema is problematic in part because it mirrors the tendency of the law to treat animals as objects, similar to mere property, versus legal subjects. Subjects in cinema and law have their own desires, needs, and preferences, which the criteria Role in Narrative and Agency are intended to reflect. Additionally, Animality points to respect for dogs’ rights to engage in natural behaviors and have their species-typical and individual needs fulfilled, areas in which the law needs to improve by not only acknowledging animal sentience but also defining what this means in practice. Language matters because referring to animals as “it” reinforces their legal status as property and obscures their status as sentient beings. Using “he/she/them” pronouns aids in “moving them from objects to subjects of their own lives” [69] (p. 393).

Below, I apply the Canine Characters Test to two examples, one television show and one film. Following this application, I identify three problematic tropes that would not meet the criteria posed in the test.

### 4.1. Applying the Test

The Canine Characters Test was borne of inspiration. While watching the TV series *Downton Abbey* [70]—as entertainment, not an object of research—I was struck by how many scenes Isis was in, and the casual and naturalistic ways she was depicted as a tertiary character. These observations provided the seeds of an idea for a test for canine characters, similar to the Bechdel Test. Around this time I happened to see the film *Knives Out* [71] in the theater. With the beginnings of this idea percolating in my mind, I found the ways the dogs in this film were portrayed to be interesting, and similar to some of the elements I noticed in Isis’s portrayal.

These two works gave rise to the idea for the Canine Characters Test, and once I developed the test criteria, I rewatched both and took extensive and detailed notes on each scene in which the dogs appeared. So, I include these works as examples, but any other film or TV show with canine characters could be used. As I developed the test, I analyzed additional works (including *Riverdale* [72], *Mad Men* [73], and *White God* [74]) but include detailed analysis of only two here, as this article is intended to introduce the test and demonstrate how it may be applied, rather than encompass a comprehensive analysis of dogs in film.

I use *Downton Abbey* and *Knives Out* to illustrate how the test may be applied, but my intention is that the test will be used by others, including myself, to analyze a greater number of works and connect them to the evolving real-world lives, social conditions, and legal status of dogs. My methodology is similar to the grounded theory approach [75] and uses inductive or “bottom-up” reasoning. This is an exploratory study and the questions raised by the test are open-ended rather than conclusive.

In the plot descriptions below, I mark in bold parentheses instances where the test criteria are demonstrated.

### 4.2. Downton Abbey

The opening credits of British historical drama *Downton Abbey* (2010) start by zooming out from the back end of a Labrador Retriever, tail happily wagging as he walks beside his guardian, Lord Grantham, patriarch of the estate. This is Pharaoh, the Crawley family’s dog in season one.

*Downton Abbey* provides an example of two canine characters, one whose representation would fail and another who would pass the Canine Characters Test. Pharaoh appeared in six of the seven episodes of the inaugural season and remained in the opening credit sequence for all six seasons, but was not developed as a character. He did not have his own storylines nor was he ever referred to by name. Pharaoh served an ornamental function in season one of *Downton Abbey*—more of a prop than a character—and does not pass the Canine Characters Test. However, the portrayal of the Crawleys’ canine family member would change dramatically in season two, when Pharaoh was replaced (without comment in the story) by a new canine character: Isis.

Isis, a yellow Lab like Pharaoh, appears in a majority of episodes in seasons two through five (60% in seasons two and three and 90% in seasons four and five). She materializes naturalistically during scenes (**Animality**), is referred to by name (**Language**), and is portrayed as a treasured family member (**Role in Narrative**), particularly beloved by central character Lord Grantham.

Isis even has a few pivotal storylines of her own, including being the subject of a kidnapping caper in the season two finale (**Role in Narrative**). The kidnap subplot—in which devious footman Thomas hides Isis in a shed so that he can later “find” her in a bid to curry favor with Lord Grantham—is effective only because of Lord Grantham’s deep affection for Isis, which had been well-established by this point through a series of brief but regular interactions conveying their bond to the audience.

The kidnapping caper is pivotal to the overall plot because it leads Lord Grantham to give Thomas a promotion, which will have many repercussions in upcoming storylines and is therefore a plot turning point that pivots on Isis and her status as a cherished member of the Crawley family (**Role in Narrative**).

Isis is portrayed as a part of the Crawley family, but has a particular bond with Lord Grantham and her appearances are often with him. These can be unexpected, seeming to occur at random. Isis is not always with Lord Grantham as he moves through scenes, but sometimes she is. These appearances are markedly realistic (**Animality**). She is often seen by his side when he walks into a room, or sitting next to him while he is at his desk or on a couch in a group scene. In some group scenes portraying multiple people sitting around talking, Lord Grantham is shown casually caressing Isis’s head as she sits next to him, completely integrated into the scene in a naturalistic way.

Isis also appears in many family scenes without comment, and without being the focal point. Often she is just “there.” These appearances are striking precisely because of their mundanity. In order for Isis to appear in scenes, she does not need to be shoehorned into plots, nor is she depicted anthropocentrically as a cipher for human values such as nobility, loyalty, or the like. However, as the story is primarily centered around the adult human characters and their stories, it is reasonable that Isis’s presence would be backgrounded. The two young children, both born during the course of the show to main characters, also appear infrequently, and less than Isis. This is due to the story revolving around the adult characters as well as the upper-class social norms of the time, in which children were cared for primarily by nannies and were less centered in family life than they are today.

Despite Isis’s presence being incidental in most (though not all) scenes in which she appears, her recurring presence continually reminds the viewer that she is part of the family too (**Role in Narrative**). Another striking element of Isis’s portrayal is its verisimilitude, with her depicted as “doglike” in quotidian ways (**Animality**). For example, in the final episode of season one, Isis appears—seemingly at random—during a scene depicting human characters playing charades in the drawing room. Apropos of nothing, Isis suddenly jumps up and barks during the game. Lord Grantham addresses her by name to shush her, saying “Isis, Isis” (**Language**). (The subtitles do not name her, and merely read: “Dog barks.”) This interaction is completely mundane and reads very much like a chance dog moment that would happen in real life during a party.

A particularly loving scene occurs in episode seven of season three, as Lord Grantham is preparing to take an ocean voyage to America. The entire family is lined up in the expansive driveway to see him off and wish him well on his journey. Isis is there with Lord Grantham’s son-in-law, Tom Branson. Lord Grantham goes down the line and says goodbye to each person in turn, reaching Tom and Isis last. He says: “Bye Tom, look after all my womenfolk, including Isis.” Then he quietly adds, “Especially Isis” (**Role in Narrative/Language**). He then leans down and, taking her face gently in his hands, kisses the top of her head and pets her ears and neck. Tom responds with an amused smile, “I’ll try my best”. Isis is shown going back into the house with the rest of the family after Lord Grantham’s car pulls away.

Isis appears again near the end of the next episode. Lord Grantham is back from his trip and strolling through a bazaar set up on the grounds of Downton Abbey and Isis is walking beside him. She was not in the episode until he returned, and even then she is not seen right away. The seeming arbitrariness of when she appears is interesting and shows she is more than a prop. If she were always shown with Lord Grantham we might interpret her as being an extension of him. However, the offhand way she comes and goes, unleashed and seemingly of her own will, gives the impression she has a life outside of the scenes in which she appears (**Agency**).

Isis has many other significant appearances throughout the series, culminating in her death toward the end of season five when the dog playing her retired due to age. Isis’s death, which is heavily foreshadowed and woven into earlier scenes over the course of the season, is treated with poignancy, gravitas, and respect. The lead-up to her death is its own subplot, with Isis subtly shown to become sick over time. Throughout the season, it becomes increasingly apparent that something is wrong with her, and some of the characters remark between scenes that she does not seem like herself. In the episode of her death, Lord Grantham and his wife stay up all night with her and bring her into their bed. She dies lying between them: a quite remarkable and respectful farewell to this tertiary character (**Role in Narrative**).

During the last episode of the sixth and final season, in addition to the resolution of numerous storylines, the family welcomes a new puppy into the home, an event that is portrayed as a cause for joy and celebration. The puppy character, named Teo, briefly appears on-screen twice in the 2019 feature-length *Downton Abbey* film as an adult dog.

In summary, Isis easily passes the Canine Characters Test. She figures prominently in the main story as a tertiary character and treasured family member, and features in at least one subplot that directs the story (**Role in Narrative**). Isis moves through her scenes at Downton Abbey unleashed and under her own volition (she is shown on leash in public spaces like the train station or walking in town next to busy roads), and the relationship between her and Lord Grantham is shown to be one of respect and affection (**Agency**). Isis is frequently referred to by name and with the pronouns “she” and “her” (**Language**) and is depicted naturalistically (**Animality**).

### 4.3. Knives Out

The comedic murder mystery film *Knives Out* (2019) revolves around skilled detective Benoit Blanc’s investigation of members of a wealthy, eccentric family for their role in the death of its patriarch, Harlan. Also central to the story is Marta, Harlan’s nurse caregiver and—shockingly to the family—the sole beneficiary of his will. It will eventually be revealed that Marta played a complicated indirect role in his death but did not murder him. The family includes two German Shepherds who, while not central characters, have multiple appearances and are integral to the twisty mystery plot in several ways.

First, the dogs did not bark when Marta crept up to the gate, allowing her to follow through on her plan to climb the trellis and into Harlan’s room—an action that is central to the plot (**Role in Narrative**). As depicted in a voiceover, Harlan tells Marta, “Come through the gate. The dogs will know you. They shouldn’t bark.” As Marta is shown approaching the gate, the dogs run up to her and greet her with friendly quiet whimpers. This scene rhymes with a later scene when the actual murderer approaches the same gate at night. The dogs do bark this time, forcing him to temporarily abandon his plan.

The dogs are also involved in foreshadowing the identity of the murderer (**Role in Narrative**). When Marta, Benoit, and the other detectives are walking through the woods looking for clues, the unleashed dogs bound up to Marta (**Agency**). She bends down to pet one of them, saying “Good boy” (**Language**). Detective Benoit remarks, “The best judge of character is a dog. I’ve always found that to be true.” This foreshadows a scene when the character of Ransom—eventually revealed to be the murderer—is introduced and the dogs playfully circle him, jumping and barking (**Animality**). He rebuffs them, protesting, “No, no, no! Hey … stop! Stop!”

The dogs, who are unnamed, become integral to the mystery at the center of the plot a second time when one of them runs up to Marta with a broken trellis piece in his mouth and drops it directly at her feet (**Role in Narrative**). This is the biggest clue, which she very much does not want to be revealed. She is able to toss the incriminating piece of wood aside while Benoit’s back is turned. However, in the next scene, one of the dogs will bring it back (**Agency**).

This is a seemingly random detail—there are many other ways the broken trellis piece could have been discovered—that weaves the dogs into the main story in a meaningful way. It shows their agency without anthropomorphizing them or endowing them with near-magical mystery-solving qualities, as in the “miraculous canine” trope discussed below. The behavior of the German Shepherd duo in *Knives Out* is thoroughly doglike, which is what makes this an interesting example of dogs depicted in film (**Animality**). They are not overly integrated into the human family and are never seen in the house. Rather, they have the sprawling grounds to freely roam about in, and are frequently shown outside (**Agency**).

Yet it is implied that they are not “outside dogs” who would function strictly as guard dogs for the grounds. At one point Benoit notes, referring to the evening of the murder: “The dogs were outside that night.” Saying they were outside “that night” implies they are not always outside. In contrast to *Downton Abbey’s* Isis, who is portrayed as embedded within the family unit, typically appearing with a human character, the German Shepherds of *Knives Out* are shown doing their own thing in the background during outdoor scenes (**Agency**). They frolic in and out of the frame and although they interact with the human characters, they are not visually tied to them (**Animality**). They have an independent subjectivity that is cinematically suggested to exist on its own. However, they are not completely separate, as shown by their importance to the plot, and they are clearly part of the family (**Role in Narrative**).

The dysfunctional family at the center of the murder investigation is not close-knit—far from it, they are exceedingly prickly and combative with one another—so it is perhaps expected that the dogs in this familial context will be more independent, in contrast to Isis, who was part of a tight-knit family unit.

Though more independent than Isis, the German Shepherds of *Knives Out* are also shown to be part of the family, as opposed to free-floating ancillary characters. They are greeted warmly by one of the main characters when they rush up to her car; they appear in a montage at the end of the film that shows a brief reaction shot of each family member when Ransom is being arrested; and one of the dogs is included in the dramatic final wide shot of the whole family (**Role in Narrative**).

In terms of characterization, the dogs are used as cinematic shorthand to boost the audience’s esteem of Marta and Benoit as presumably good people. Both characters display kindness to the dogs. Marta pets and praises them, and in one scene as Benoit absentmindedly fidgets with a ball, he notices one of the dogs is keenly interested, addresses the dog directly (“You want this?”), and then tosses it for him. In contrast, Ransom, who is also rude to the human characters, rejects the dogs in annoyance when they approach (“No, stop!”). They also jump on him more than the other characters, perhaps sensing his dislike for them, or conveying their own dislike of him with deliberate attempts to annoy him.

The German Shepherds of *Knives Out* have nine relatively brief appearances, but they bookend the film, appearing in both the opening and closing shots. The opening shot depicts the dogs running in slow motion toward the camera, the stately mansion in the background. In the final minutes of the film, as the police lead Ransom away in handcuffs, the dogs casually move across the frame. Moments later, they cross the frame again in the other direction, with one of the dogs doubling back, a movement that looks markedly naturalistic (**Animality**).

In a second shot of Ransom, he fades to a blur, but the dogs are still in focus. Preceding this climactic moment, several other characters are shown reacting as Ransom is taken into custody. The dogs are portrayed as oblivious to the high drama happening around them. Either that, or they do not care because he is a “bad” character, Ransom’s dislike of the dogs—and likely theirs of him—having been suggested in earlier scenes. (Regarding their obliviousness, I commented in my notes: “As they should be! A ‘miraculous canine’ might growl and bite his pants as he is being led away.”) The way the dogs are portrayed in this dramatic denouement is distinctly realistic, and thus easily passes the portion of the Canine Characters Test concerned with the depiction of natural behaviors (**Animality**).

Regardless, the ultimate message of this scene is that the German Shepherd duo is part of the family. This is clearly conveyed by the dogs’ inclusion in this penultimate scene, in which every family member is shown at least briefly. Lastly, we come to the dramatic final shot, framing Marta on the balcony as the whole family looks up at her. When the camera zooms out, one of the dogs is shown sitting with them, visually conveying his inclusion in the family (**Role in Narrative**).

In summary, the German Shepherd duo mostly pass the Canine Characters Test. Their actions direct the story in a meaningful way and they are shown to be part of the family (**Role in Narrative**). They are shown displaying agency and seem to live an unencumbered day-to-day life on the sprawling grounds (**Agency**). The dogs are not referred to by name, but neither are they referred to as “it.” They are called “boy” (as in “good boy”) at times and are spoken to, as well as about (**Language**). They are portrayed naturalistically (**Animality**). Thus, they clearly fulfill three of the four criteria. The Language category is a bit more ambiguous. One assumes the dogs have names, even if they are not used on-screen. But this illustrates the relatively background nature of these canine characters, even as they emerge a couple of times to meaningfully direct the story with their thoroughly doglike behavior.

## 5. Problematic Tropes

Vanishing, ornamental, and miraculous canines are just a few examples of problematic portrayals that would fail the Canine Characters Test. A vanishing canine disappears from the plot with no explanation. It would be difficult for them to pass the Canine Characters Test due to their not having a role in the narrative once they vanish. Ornamental canines serve as props more than characters and miraculous canines embody positive stereotypes that are inauthentic.

### 5.1. The Vanishing Canine

Vegas the yellow Labrador Retriever lives with teen-aged Archie Andrews and his dad Fred on the teen drama mystery *Riverdale* (2017). Vegas appears sporadically over the course of the first three seasons and is shown to be a loved member of the Andrews family, but he inexplicably disappears from the show in season four. *Riverdale* became increasingly untethered from reality as the seasons progressed and, as the show became more bizarre, juggling chaotic and unrealistic subplots, there just may not have been room for Vegas. However, the way Vegas appears in certain scenes in the family home but is absent from other, very similar, scenes is curious. While Isis’s appearances on *Downton Abbey* were unpredictable—my viewing notes are replete with surprised comments about her popping up here or there in a scene unexpectedly—they were more regular and hence felt less arbitrary. In addition, Isis lives in a mansion on a sprawling estate and could be off in any number of places when she is not on-screen. Vegas lives in a modest suburban home. Where is he, when he is absent?

Sometimes television shows introduce a baby or young child only to have them virtually disappear for the remainder of the series. Children and babies vanish from storylines for a variety of reasons, including difficulties around laws pertaining to minors on set and the problem of young children aging faster than their on-screen characters.

Canine characters may vanish for similar reasons but the trope of dogs disappearing from stories after being introduced unfortunately reflects a societal problem: many dogs are obtained only to be abandoned at a later time (in cases of relinquishment to a shelter, other rehoming process, or worse). Dogs are often figuratively abandoned as well, in the case of their basic needs for companionship, mental stimulation, and exercise being neglected—perhaps after a short period of excess attention when the dog’s presence in the home is novel.

It is problematic when dogs appear once or twice never to be seen again because it mirrors the dominant culture, which treats dogs as expendable despite paying lip service to their being family members. According to the Shelter Animals Count national database, 360,000 dogs were killed in U.S. shelter facilities in 2023. This number has increased in recent years, with “a notable rise in non-live outcomes for dogs (+24% or 78,000 more dogs from 2022; +64% or 157,000 more dogs from 2021). Additionally, non-live outcomes for dogs have risen by 12% (42,000 dogs) compared to 2019” [76] (p. 10).

Shelters across the country are full, and are now entering their fourth year of having too many animals and not enough adoptions—especially for dogs. Because many shelters and rescues are operating at- or over-capacity, the number of surrenders and overall intake is likely lower than it would be if space were available.

[77] (n.p.)

As I have argued, animals’ legal status as property reinforces their cultural status as expendable, in contrast to their contradictory social construction as family members. The role of companion animals in society is in flux, and different types of portrayals on-screen reflect this instability. While undoubtedly not intentional on the part of creators, vanishing cinematic canines unfortunately reflect social conditions that are detrimental to dogs’ wellbeing, telegraphing that dogs are disposable, or at least marginal, rather than embedded in the family unit.

Vegas mostly passes the Canine Characters Test—he is shown displaying agency, his behavior is authentically doglike, and he is spoken of affectionately by name—but while he is featured in a few pivotal scenes, his portrayal falls short for Role in Narrative. More problematic than simply not figuring prominently in the story is that he vanishes—not appearing enough to even become an ornamental character.

At the time of this analysis, only seasons one through four of *Riverdale* had been released, but Vegas’s disappearance is finally addressed in season five when in a bit of retconning it is revealed that Vegas went to live with Archie’s mom in Chicago when Archie enlisted in the military at the end of season four, and later died while Archie was overseas. Archie says, “He was my best friend”, even though Vegas was missing for all of season four. But this acknowledgment (prompted by a character asking Archie, “Hey didn’t you used to have a dog?”) serves to introduce a new shelter dog character. 

Vegas gets another—quite meaningful—appearance in season six when Archie is in an afterlife scenario called “the sweet hereafter”, which is a heaven individually tailored to each character. In this personal heaven, we see Archie’s love interest Betty, their future children, and Vegas. When Veronica journeys to the sweet hereafter to tell Archie he must return to the “real Riverdale”, Archie protests, saying: “This is the real Riverdale. Everything I’ve ever wanted … is all right here.” And on that beat, Vegas enters the scene with a happy bark. Three seasons after disappearing from the show, he is brought back for a scene depicting Archie’s ideal heaven, which is a sweet nod to his importance to the Archie character, despite vanishing from the story.

### 5.2. The Ornamental Canine

Sometimes the dog does not disappear altogether but becomes an ornamental character, dusted off for particular scenes as part of the set design, usually to represent a visual theme. This “ornamental canine” appears as part of the mis-en-scene and is essentially a prop, often used to signify traditional family or the private interior world of the home versus public life. As Horowitz notes: “In films the use of dogs as prop, rather than as dog, continues apace. As punctuation to a scene, to move the plot forward, to set a character’s or environment’s tone, dogs are used as symbols” [37] (p. 233).

The golden retriever Polly in *Mad Men* (2007) is an example of an ornamental canine. Don Draper brings Polly home as a Christmas present for his children in season one to distract from his unexplained absence for much of the day. From her introduction, she represents the home life that Don is systematically destroying even while trying to preserve a veneer of normalcy and traditional family values. To Don in the world of *Mad Men*, Polly also serves as a prop, similar to the proverbial white picket fence—an essential element of the portrait of idealized affluent white suburban family life of the early 1960s.

Polly never disappears entirely from the story but is absent from many at-home scenes where a previously introduced suburban resident dog would be expected to be seen. But just when the audience may have forgotten that the Drapers have a dog, she suddenly appears, shown with the children or in another way to visually signify “family” to the viewer as a stark juxtaposition to protagonist Don’s public life of business and serial infidelity outside the home.

Along with the children, Polly represents home, hearth, and traditional marriage. Unlike the older child, Sally, but similar to the younger child Bobby, Polly does not have storylines of her own. The two Draper children and Polly the dog exist on a character continuum, with Sally Draper being the most developed, Polly the dog being the least developed, and Bobby Draper somewhere in the middle. Bobby is almost, but not quite (as he displays some agency and acts as a foil to his sister) as ornamental as Polly.

As with young children, it is an added complication to have animal actors on set, which may explain some of the “vanishing syndrome”, a continuum that includes the ornamental canine. However, to support the elevation of companion animals’ legal and cultural status, works of popular culture should present them as truly integrated into the family. This does not mean they need to be on-screen all the time or even have their own subplots—in a show where the main characters are human, it is enough for the dog to be realistically portrayed as woven into the lives of these characters.

### 5.3. The Miraculous Canine

Another example that would fail the Canine Characters Test is if the dog were portrayed as a “miraculous canine.” This is a common theme in characterizations where the dog is central to the plot (e.g., Lassie in some renditions). The miraculous canine is similar to the “magical Negro” trope identified and criticized by film director Spike Lee, which refers to a Black supporting character who comes to the aid of White protagonists in a film and often possesses special insight or mystical powers [78]. This trope is also the subject of a 2024 satirical comedy film, *The American Society of Magical Negroes*.

The miraculous canine is amazingly intelligent, perceptive, and courageous. This dog selflessly helps their humans (or kittens in a burning building in 1978’s *The Magic of Lassie*), preternaturally senses danger, and performs feats of incredible bravery, exceptional physical skill, or cognitive genius. They are often depicted heroically and sometimes sacrifice themselves for humans. While a noble portrayal, it is unrealistic, and would not meet the “animality” criterion of the Canine Characters Test.

Of course, movies and TV shows do not have to be realistic, and characters can behave in unbelievable ways even within non-fantastical genres. Fantastical representations can also be potentially empowering. But unrealistic depictions become problematic when they reflect sexist, racist, or speciesist stereotypes.

As discussed above, these idealized portrayals of dogs serve an ideological function—particularly when they reinforce cultural myths that are harmful to dogs, e.g., that they are “little people in fur coats” (and should behave accordingly) or that they exist to serve human needs and desires, such as providing “unconditional” love. However, idealized portrayals may also contain seeds for a counter-hegemonic, pro-animal vision. To mindfully share one’s life with a nonhuman animal can be an awe-inspiring, if not technically miraculous, experience—not for the superpowers they possess, but for who they actually are.

The mundane miracle of being in the company of a dog can at times be glimpsed in cinematic works that pass the Canine Characters Test. When grounded in animality, “miraculous” traits reflect the real world and the individual dogs within it. When animality is discarded, these traits transcend reality in ways that reflect speciesist ideology, similar to the way the magical Negro is a racialized trope.

## 6. Discussion: Impacts of Film

As the Frankfurt School critical theorists well understood, products of mass culture are instruments of social reproduction [3]. Popular culture helps to structure our ideas about ourselves and others—including animals. Portrayals of dogs in film and television frame our relations with and subconscious attitudes about them, even as those relations and attitudes, in turn, inform on-screen portrayals.

In contrast to works that foreground canine characters as major protagonists, the meaning of depictions in which they are minor characters is less obvious but perhaps even more important. These naturalized portrayals of the dog as woven into family life, but not centered as a main character, appear more realistic and hence are a Trojan horse of potential meanings that can work on viewers’ subconscious minds precisely because they are less conspicuous.

In considering the Canine Characters Test and representation, we can hypothesize that films and TV serve a social modeling function, and thus, it is important to see respectful relationships with, and treatment of, dogs reflected on-screen. Though one film is unlikely to singlehandedly trigger large-scale social changes, it can act as a prismatic condensing symbol that boosts latent norms percolating below the surface of the dominant culture—with the right catalyst or catalysts, these norms can become ascendant.

We can further hypothesize that an accumulation of works that pass the Canine Characters Test will see corresponding improvements in the real-world lives of dogs and significant shifts in their currently ambivalent cultural status, and by implication their legal status (due to the recursive relationship between law and culture).

Evidence supporting the real-world impacts of cinematic representations of animals is mixed. Despite its importance to culture and socialization, it is notoriously difficult to measure the effects of pop culture on attitudes, beliefs, and behavior. Little empirical work has been conducted on audience responses to animal imagery [28], with some notable exceptions.

In those cases, at times the purported effects of specific animal films have been revealed to be more myth than fact. Widely reported phenomena like the “Jaws effect”, “Bambi syndrome”, and “Finding Nemo effect” have variously been found either to be nonexistent, exaggerated, or, at best, considerably more complex than presented in popular media [79,80,81,82,83,84,85,86]. Despite the enduring popularity and intuitive appeal of these concepts, supportive evidence has been scant.

However, in a chapter entitled “Would Bugs Bunny have Diabetes?: The Realistic Consequences of Cartoons for Non/Human Animals,” George makes a connection between the carrot-loving character Bugs Bunny and chronic illness in rabbits, due to erroneous assumptions about what constitutes a nutritious diet based on this cartoon [87]. Prompted by several reports of neglect, the Royal Society for the Prevention of Cruelty to Animals launched a public awareness campaign called “What Bugs a Bunny” to combat widespread misperceptions about rabbit care, which were revealed in surveys, including beliefs that “rabbits should eat carrots because that’s what Bugs Bunny does”, when the truth is real rabbits “shouldn’t be eating carrots too often” [87] (p. 67).

In addition, the impact of movies on dog breed popularity has been empirically documented [88] and public crazes for purebred dogs have followed the release of popular films and TV shows featuring that type of dog—or in the case of the CGI dire wolves of *Game of Thrones* (2011)*,* the presumed next best thing: Siberian huskies.

Rescue groups and shelter facilities reported receiving an influx of surrendered Siberian huskies during and after the run of HBO’s massively popular television series, which featured fictional dire wolves, a species that has been extinct for thousands of years but resembles northern dog breeds such as huskies—a notoriously high-maintenance breed due to their intelligence, propensity for boredom, and need to run. In addition to timing, shelters were able to connect these abandoned huskies to the *Game of Thrones* fad because many were named after characters, both lupine and human, from the show [89,90,91,92].

Likewise, a sharp increase in the number of unwanted Dalmatians was reported after the release of Disney’s remake of the movie *101 Dalmatians* (1996) [93] and a craze for German Shepherds followed canine film star Rin Tin Tin’s immense popularity in the 1920s, which resulted in unscrupulous and cruel breeding practices [94].

### 6.1. Challenging the Human Gaze?

Classical sociological theory via symbolic interactionism taught us that only humans could communicate in a way that enabled “taking the role of the other” [95] and thus engage in meaningful social interaction. The emergent field of animal studies has challenged this conventional sociological wisdom on many fronts over the last few decades [96,97,98,99]. Outdated beliefs about animals, communication, and culture are also increasingly being challenged from both scientific and political perspectives [100].

Setting aside outmoded ideas about animal subjectivity, it is difficult, if not impossible, to fully transcend the human gaze in film and television given the creators themselves are human. However, as with the similarly complex questions that arise in the context of lawyers representing animals, “the idea that we can retreat into our own isolated subjectivity and refrain from speaking for or about others is naïve” [101] (p. 28). In the context of cinema, perhaps transcendence could be achieved if the entire work were filmed from the vantage point of an unobtrusive camera attached to a dog’s collar (though this technique would likely not produce enough footage or dramatic tension to sustain an entire movie or television program with widespread appeal). However, creators can and do surpass their own viewpoint and attempt to portray the perspective of others when they create works of art. This is indeed one of the functions of art and pop culture: to present fresh and unique perspectives, to take us outside of ourselves, and to let us inhabit the social world of another, if just for a short time.

These “others” are usually human, but not always. As problematic as it is to attempt to “speak” for animals through art, it can be done respectfully, attentively, and with the implicit understanding that the process is interpretive and creative, yet still grounded in reality—in what we know about the lives of animals. Filmmakers and writers who portray animal characters would do well to make sure they are well-informed about the animals they are depicting, as one way to temper the anthropocentric gaze. 

This does not mean that animals can never be portrayed fantastically or unrealistically. Heroic but unrealistic characters can challenge traditional cultural stereotypes and regressive film tropes. For example, the critically acclaimed TV show *Buffy the Vampire Slayer* (1997) broke ground by portraying a teenage girl as a heroic protagonist with supernatural powers. Through its fantastical portrayal of a powerful adolescent girl, *Buffy the Vampire Slayer* upended genre conventions regarding gender and gave audiences a new icon of strength, courage, and heroism typically reserved for male protagonists [102].

Films and television shows featuring animals as protagonists may serve a similar function, but in my view this always must be balanced with an understanding of the relative powerlessness of animals in society compared with humans. Sarah Michelle Gellar had a choice and a voice in portraying Buffy. Canine performers rely completely on the writers and their handlers to represent them and are dependent on their guardians to safeguard their interests.

This does not mean that dogs lack voices or the ability to be co-subjects in interaction. However, given the stark power imbalance between canine actors and the humans who control virtually every aspect of their lives, great care and attention will need to be given by producers and caregivers to create conditions where intersubjectivity can flourish—on and off the screen. The Canine Characters Test provides one new tool to begin this project.

### 6.2. Future Directions

A content analysis of top-grossing movies featuring canines as secondary or tertiary characters in a family context would yield interesting theoretical insights—similar to research that applied the Bechdel Test to the highest-grossing movies of the past four decades [103]—as would interviews with producers and writers about the decision to include canine characters in film and TV where they are not protagonists but feature prominently enough to pass the Canine Characters Test.

Although it looked at films relevant to marginalized human communities and not animals, a 2020 report by the Center for Scholars and Storytellers at UCLA, in partnership with the Creative Artists Agency (CAA), found that films that scored high in authenticity did better at the box office. Researchers expanded this study in 2021 to encompass a total of 1000 films, analyzing expanded questions around authenticity and representation of a greater number of marginalized groups, including whether the films avoided harmful stereotypes/tropes and how well the film increased the complexity of a general audience’s understanding of the culture or group they represented [104]. This general research design could be adapted to analyze animals in film, or dogs in film specifically, and whether audiences’ understanding increased based on authenticity in representation.

Linking positive portrayals of canine characters to changes in social norms, attitudes, and behavior over time also would be a fruitful area of theoretical inquiry—in particular, drawing connections between representations of animal subjectivity and agency on-screen with concrete reforms in culture, law, and policy resulting in improved treatment, status, and well-being of dogs in society—as well as the reverse: how improvements in animals’ status in society and law may be reflected in cinematic trends over time. Finally, a comprehensive analysis of dogs in film should consider the material conditions of the dogs themselves as worker–actors and their treatment on and off set.

## 7. Conclusions

Film and television have the potential to illuminate and challenge existing power relations, showing us alternative possibilities. As such, they are critical sites of negotiation, interpretation, and meaning construction—all of which are essential aspects of social change. Specifically, culture products inform the animal rights movement’s efforts to transform dominant forms of human–animal relations—from relationships rooted in dominance and subjugation to approaches based on respect for difference and mutual cooperation. Pop culture such as movies and television—along with toys, advertisements, magazines, and popular music—is an important part of socialization, which continues through the life course, and as such must be taken seriously by social scientists and animal advocates alike. Against the backdrop of a society in which their legal and cultural status is evolving, the Canine Characters Test gives us a new lens through which to view the representation of dogs on film.

## Data Availability

Data are contained within the article.
